# Nanostructured Hydrogels of Carboxylated Cellulose Nanocrystals Crosslinked by Calcium Ions

**DOI:** 10.3390/gels10120777

**Published:** 2024-11-28

**Authors:** Alexander S. Ospennikov, Yuri M. Chesnokov, Andrey V. Shibaev, Boris V. Lokshin, Olga E. Philippova

**Affiliations:** 1 Physics Department, Lomonosov Moscow State University, 119991 Moscow, Russia; ospennikov@polly.phys.msu.ru (A.S.O.); shibaev@polly.phys.msu.ru (A.V.S.); 2 National Research Center “Kurchatov Institute”, 123182 Moscow, Russia; chessyura@yandex.ru; 3 Chemistry Department, Karaganda E.A. Buketov University, Karaganda 100028, Kazakhstan; 4 A.N. Nesmeyanov Institute of Organoelement Compounds, Russian Academy of Sciences, 119991 Moscow, Russia; bloksh@ineos.ac.ru

**Keywords:** cellulose nanocrystals, nanorods, rheological properties, hydrogels, crosslinking

## Abstract

Bio-based eco-friendly cellulose nanocrystals (CNCs) gain an increasing interest for diverse applications. We report the results of an investigation of hydrogels spontaneously formed by the self-assembly of carboxylated CNCs in the presence of CaCl_2_ using several complementary techniques: rheometry, isothermal titration calorimetry, FTIR-spectroscopy, cryo-electron microscopy, cryo-electron tomography, and polarized optical microscopy. Increasing CaCl_2_ concentration was shown to induce a strong increase in the storage modulus of CNC hydrogels accompanied by the growth of CNC aggregates included in the network. Comparison of the rheological data at the same ionic strength provided by NaCl and CaCl_2_ shows much higher dynamic moduli in the presence of CaCl_2_, which implies that calcium cations not only screen the repulsion between similarly charged nanocrystals favoring their self-assembly, but also crosslink the polyanionic nanocrystals. Crosslinking is endothermic and driven by increasing entropy, which is most likely due to the release of water molecules surrounding the interacting COO^−^ and Ca^2+^ ions. The hydrogels can be easily destroyed by increasing the shear rate because of the alignment of rodlike nanocrystals along the direction of flow and then quickly recover up to 90% of their viscosity in 15 s, when the shear rate is decreased.

## 1. Introduction

Cellulose nanocrystals (CNCs) represent a new fascinating type of nanomaterial that is derived from cellulose, the most abundant polymer on Earth. In nature, cellulose chains are organized into microfibrils that consist of amorphous and crystalline domains [1]. CNCs are usually produced by acid hydrolysis of the amorphous domains, leaving rodlike nanocrystals (4–70 nm in width and ∼100–500 nm in length [2]). Within CNCs, the cellulose molecules are arranged parallel to each other with a helical twist and are held together by hydrogen bonds [3]. Most often, sulfuric acid is used for CNC preparation. It introduces negatively charged sulfate half-ester groups onto CNC surfaces. These groups create an electrostatic repulsion, which stabilizes the suspensions of nanocrystals in water. Negatively charged CNCs can also be prepared by TEMPO (2,2,6,6-tetramethylpiperidine-1-oxyl)-mediated oxidation of CNCs, providing carboxylate groups on the surface of nanocrystals [4].

CNCs are gaining increasing interest in the fields of materials science and nanotechnology due to their unique properties. Being produced from a renewable inexhaustive natural source, CNCs are environmentally friendly, biocompatible, and biodegradable. They have high crystallinity (54–88%) [5], high specific surface area (800 m^2^/g) [6], and high strength (elastic modulus of 110–220 GPa, tensile strength of 7.5–7.7 GPa) [7].

CNCs are often used as bio-based nanofillers for preparation of polymer nanocomposites [8,9,10], but they can also form materials themselves, for example, colloidal gels. These gels are based on self-supporting percolated networks of CNCs that are stabilized by hydrogen bonds between nanocrystals. CNC gels can be formed without any additives even though the nanocrystals are similarly charged, but the gelation requires a rather high concentration of nanocrystals (above 12 vol% [11]). Recently, Lewis et al. [12] proposed to use freeze–thaw cycling to prepare CNC gels. In this case, the physical confinement of CNCs between the growing ice crystal domains induced the aggregation of nanorods strengthening the gels. As a result, the freeze–thaw cycling increased the complex modulus of CNC suspensions by more than two orders of magnitude, from 0.4 to 150 Pa at rather low CNC concentration (4 wt%) [12]. Later, Li et al. [2] applied sequential freeze–thaw cycling coupled with hydrothermal treatments to further enhance the complex modulus of CNC gels up to 160 to 32,000 Pa. Also, they used freeze-drying of the hydrogels to obtain lightweight CNC aerogels with Young’s modulus of 0.114–3.98 MPa.

Thus, the preparation of CNC gel without additives requires either a high concentration of nanocrystals or a sophisticated treatment. Most common strategies for preparing CNC gels are based on a reduction of electrostatic repulsion between similarly charged CNCs by adding salt. By suppressing the repulsive barrier between the particles, salt allows attractive interactions such as van der Waals forces and hydrogen bonding to dominate. The CNC gelation can be induced by different salts [13,14,15]. For instance, Shafiei-Sabet et al. [13] prepared the CNC hydrogels by NaCl addition. But most often, multivalent ions are used to produce CNC gels. Chau et al. [14] studied the CNC hydrogels formed at 4 wt% CNCs in the presence of different multivalent cations (Ca^2+^, Mg^2+^, Sr^2+^, Al^3+^). It was observed that hydrogel stiffness increased with increasing charge number and ionic radius of the cations. Unexpectedly, the enhancement of the gel stiffness was accompanied by an increase in mesh size instead of its decrease. The authors attributed this effect to side-by-side aggregation of CNC nanorods induced by added cations, which made the network stiffer [14]. For instance, the increase in the complex modulus values |G*| upon increasing charge number of cations in the range of Na^+^ → Mg^2+^ → Al^3+^ was explained by the formation of stronger and thicker fibrillar aggregates [14], although this suggestion was not confirmed by structural studies. The observed increase in |G*| values upon increasing the ionic radius of divalent cations Ca^2+^, Mg^2+^, Sr^2+^, at the same Debye length, permitted the authors to suggest that not only electrostatic screening contributed to this effect, but also the crosslinking of different nanocrystals by added cations [14]. At the same time, in another study [15] it was shown that charge screening effects is the main reason for the enhancement of rheological properties of CNC suspensions by salt since the maximum strength of CNC gel was the same independently of the valency of added salt ions. These contradictions require further studies to reveal the real reasons for the CNC gels’ strengthening by multivalent cations.

CNC gelation, which is noncovalent, has many advantages due to its reversibility. The reversibility means that the disrupted gel structures can spontaneously recover, which makes it useful for several applications, where the recovery of the gel structure after its extrusion or injection is required, for instance, in 3D extrusion printing [16], preparation of injectable hydrogels for medical use [17], or thickening fluids for the oil industry [18,19]. However, this phenomenon in CNC gels has not been widely studied and more research is needed to fully understand its potential and the factors that affect the recovery time and completeness.

The present paper aims to study the rheological properties of hydrogels formed by carboxylated CNCs in the presence of calcium salt CaCl_2_. Calcium salt was chosen among different multivalent metal salts because of its biocompatibility [20]. The study focuses mainly on two aspects: (i) revealing the interaction between divalent calcium ions and carboxylic groups of the nanocrystals and its effect on the rheological properties and structure and (ii) exploring the reversibility of the gel formation. The research clearly demonstrates that calcium ions not only shield the electrostatic repulsion, but also crosslink the nanocrystals. The crosslinking of the gels is reversible, as they can be broken down by shear forces and then quickly regain up to 90% of their original viscosity at lowering shear rate, which is highly desirable for various applications.

## 2. Results and Discussion

### 2.1. Phase Behavior

In the present study, the experiments were performed at a fixed concentration of CNCs equal to 3 wt%. These aqueous CNC suspensions are quite stable: they do not exhibit phase separation for at least a few months. This may be due to the high charge of CNCs, so their segregation in a separate phase will lead to increased electrostatic repulsion and a significant loss of translational entropy of counterions. The content of carboxylic groups on the surface of nanocrystals under study is equal to 1.2 mmol per gram of CNCs. At pH 6.5, used in our experiments, all carboxylic groups in the CNC suspensions appear to be deprotonated, considering that the pK_a_ value should be close to 3.9, as reported for carboxylated cellulose nanofibers [21]. When all carboxylic groups are deprotonated, the surface charge density of CNCs can be estimated as ca. 1.3 e/nm^2^ (see the Materials and Methods section for details), which is a relatively high value.

Upon addition of CaCl_2_, the system undergoes gelation and remains homogeneous until the salt concentration reaches 0.75 mM. At CaCl_2_ concentrations higher than 0.75 mM, one can observe syneresis—a gel shrinking with the expulsion of solution on the top of the gel. In the case of ordinary polymer gels, such expulsion of the solution was suggested to occur when the number of crosslinks exceeds the natural number of interpolymer contacts in the semidilute solution [22]. Probably, a similar explanation is valid when instead of polymer chains polymer nanocrystals form a gel.

### 2.2. Salt-Induced Gelation

**Figure 1:**
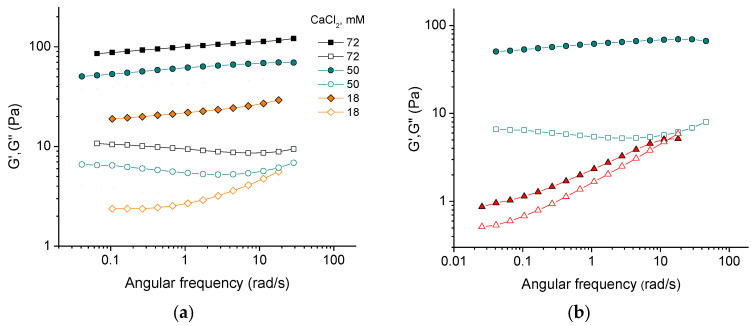
(**a**) Frequency dependencies of storage G′ (filled symbols) and loss G″ (open symbols) moduli for suspensions containing 3 wt% CNCs and different concentrations of CaCl_2_; (**b**) frequency dependencies of storage G′ (filled) and loss G″ (open) moduli for 3 wt% suspensions of CNCs with 50 mM CaCl_2_ (circles) and with 150 mM NaCl (triangles), providing the same ionic strength.

Figure 1a demonstrates the frequency dependencies of the storage G′ and loss G″ moduli of 3 wt% CNC suspensions at different concentrations of CaCl_2_ salt. For all the samples under study, the storage modulus G′, which characterizes the elastic response, is higher than the loss modulus G″, which characterizes the viscous response, over the entire range of studied frequencies (from 0.04 to 30 rad/s). This indicates the gel-like behavior of all systems, since the elastic behavior dominates over the viscous one. At the same time, the values of G′ and G″ depend essentially on the oscillatory frequency, especially at low salt concentrations. This suggests the dissipation of energy occurring due to the motion of CNC network fragments [14]. As the salt concentration increases, the frequency dependence becomes less significant, indicating that the strengthening of the network reduces the displacement of its components.

**Figure 2:**
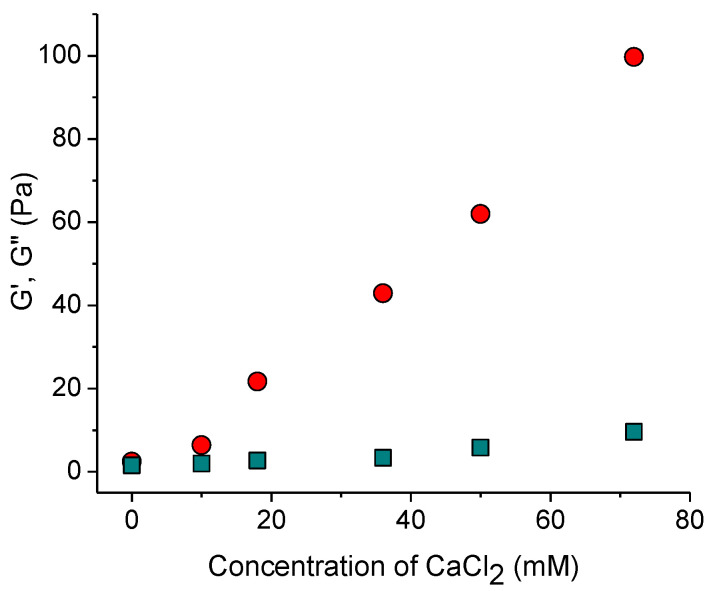
Storage modulus G′ (circles) and loss modulus G″ (squares) at the oscillatory frequency of 1 rad/s as a function of CaCl_2_ concentration.

With increasing content of added salt, both elastic and viscous contributions to elastic modulus increase (Figure 1a and Figure 2). This is consistent with the data for sulfonated CNCs [14] and carboxylated cellulose nanofibers [23]. Two reasons can be responsible for this. First, crosslinking of negatively charged nanorods by divalent cations [14,24]. Second, stronger screening of electrostatic repulsion between nanorods by salt, which makes dominant attractive forces (e.g., van der Waals interaction and/or hydrogen bonding) favoring CNC association, according to the Derjaguin–Landau–Verwey–Overbeek (DLVO) theory. Bertsch et al. [15] suggested that the second reason should dominate, since the maximum strength of CNC gel is the same independently of valency of added salt ions. But in the present system, the dynamic moduli upon addition of CaCl_2_ are by more than an order of magnitude larger than in the presence of NaCl at the same ionic strength (Figure 1b) implying that divalent cations crosslink CNCs. Density functional theory calculations have revealed that the crosslinking of carboxylated cellulose fibrils by Ca^2+^ ions is due to electrostatic interactions [25]. This should also be true for CNCs. Thus, a pronounced increase in the storage modulus with increasing CaCl_2_ concentration (Figure 2) appears to be due to the crosslinking of negatively charged CNCs by divalent calcium cations through electrostatic interactions.

**Figure 3:**
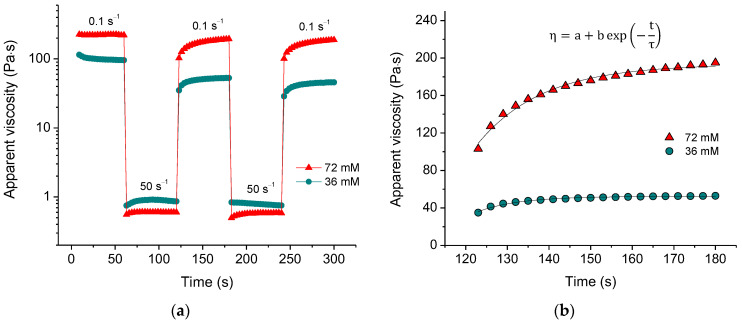
(**a**) Viscosity recovery after periodic variation of shear rate (50 s^−1^ for 60 s, 0.1 s^−1^ for 60 s, etc.) for suspensions containing 3 wt% CNCs and 36 mM (green) and 72 mM (red) of CaCl_2_; (**b**) fitting of the viscosity recovery with the exponential function for the second cycle of periodic variation of shear rate for 3 wt% CNC suspensions with 36 mM (green) and 72 mM (red) of CaCl_2_. In the formula, η is the apparent viscosity, t is time, τ is the recovery time, a,b are coefficients.

The formation of a physical, self-assembled CNC network should be reversible. To verify this, we measured the recovery of viscosity over time after a sudden change in the applied shear rate (Figure 3a). When the shear rate was increased to 50 s^−1^, the viscosity dropped by more than two orders of magnitude. However, when the shear rate was decreased to 0.1 s^−1^, the viscosity recovered. The fast decrease in viscosity with increasing shear rate indicates that the percolation network of nanocrystals was disrupted, and we observe the viscosity of CNCs aligned in the direction of the flow. The quick recovery suggests that the CNC network can be rapidly rebuilt.

The recovery curves (Figure 3b) can be fitted with an exponential function, allowing us to determine the recovery time τ. It was found (Table 1) that at low salt concentration (18 mM), the recovery time τ is rather long but then decreases significantly passing through a minimum with increasing salt concentration. This indicates that there is some optimum concentration of multivalent ions providing faster recovery. In the present system, it is close to the equimolar Ca^2+^/COO^−^ ratio. At lower concentrations of salt, the recovery is slow because of the deficiency of ions with respect to fixing the recovered structure. At high salt concentrations, more crosslinks should form, and the rebuilding takes a somewhat longer time. Table 1 shows that the recovery time in the third cycle is greater than in the second cycle, implying that it is more difficult to reconstruct the structure of the system that was recently rebuilt.

The recovery is not complete, as the apparent viscosity of the rebuilt network is slightly lower in each subsequent cycle (Figure 3a). Table 1 shows that the percent recovery significantly increases with increasing crosslinker concentration, reaching approximately 90% at 72 mM CaCl_2_. This suggests that a higher amount of calcium crosslinks promotes a more complete network reconstruction.

### 2.3. Morphology

**Figure 4:**
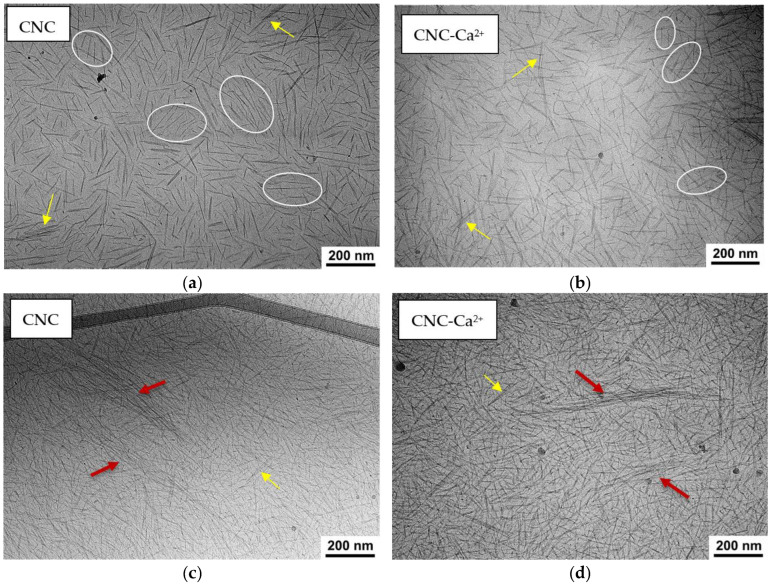
(**a**–**d**) Cryo-EM images of 3 wt% CNC suspensions before (**a**,**c**) and after addition of 50 mM CaCl_2_ (**b**,**d**) for thinner (**a**,**b**) and thicker (**c**,**d**) samples. Some domains containing CNCs oriented parallel to each other are marked by ovals. Bundles are marked by yellow arrows, fibrillar-like aggregates are marked by red arrows.

To reveal the native structure of the hydrogels under study, cryogenic electron microscopy (cryo-EM) was used. In a salt-free suspension (Figure 4a), it is possible to observe many domains in which several rodlike nanocrystals (3–7 in number) are aligned parallel to each other. This alignment is not related to the preparation method (blotting), as in the case of blotting-induced alignment, all CNCs would be aligned in the same direction [26]. In our experiment, the orientations of the rodlike CNCs in different domains are not correlated. Most likely, the parallel orientation of a few adjacent nanocrystals within the domain is due to electrostatic repulsion between the strongly charged CNCs. When salt is added, the orientation of the nanocrystals becomes more disordered, and the number of parallel-oriented CNCs within the domains decreases to only 2–3 (Figure 4b). This is because the addition of salt enhances the screening of electrostatic repulsion. In a more disordered system, nanocrystals can more easily form a percolation network required for gel formation. A similar situation was observed in cryo-EM studies of carboxylated cellulose nanofibrils [27]: some ordering in the absence of salt and a more random orientation upon addition of 0.1M NaCl, which was explained by the screening of electrostatic repulsion by added salt.

**Figure 5:**
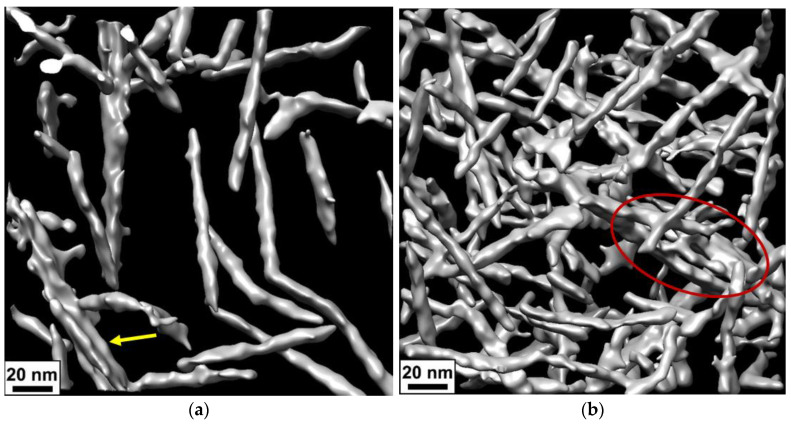
(**a**,**b**) Three-dimensional surface-rendered views of the arrangement of CNCs inside the network before (**a**) and after addition of 50 mM CaCl_2_ (**b**) obtained from cryo-ET. The bundle is indicated by a yellow arrow, and a fragment of the fibrillar-like aggregate is marked by the red oval.

On the cryo-EM micrographs, one can observe two types of aggregates formed by CNCs: short bundles of 2–3 nanorods parallel stacked with each other (Figure 4a,b) and long and thick fibrillar-like aggregates (Figure 4c,d). The latter are only seen in thick samples. In Figure 5, which was obtained using cryo-ET, one can see how these aggregates appear in three dimensions.

**Figure 6:**
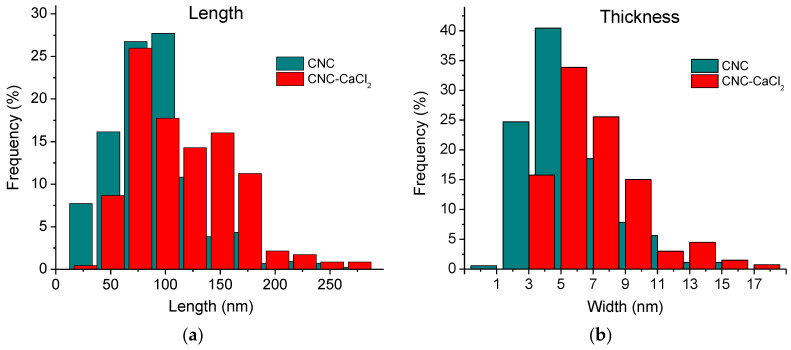
Histograms of distribution of the length (**a**) and thickness (**b**) of individual CNCs and their aggregates in 3 wt% CNC suspensions before (green) and after (red) addition of 50 mM CaCl_2_.

Both types of aggregates can be observed both before and after the addition of salt (Figure 4c,d). But the histograms (Figure 6) show that the length and thickness distributions of nanorods are shifted to larger values upon addition of salt. Longer and thicker aggregates may contribute to the gel strengthening that occurs when CaCl_2_ is added.

### 2.4. CNC-Ca^2+^ Interactions

#### 2.4.1. Isothermal Titration Calorimetry (ITC)

**Figure 7:**
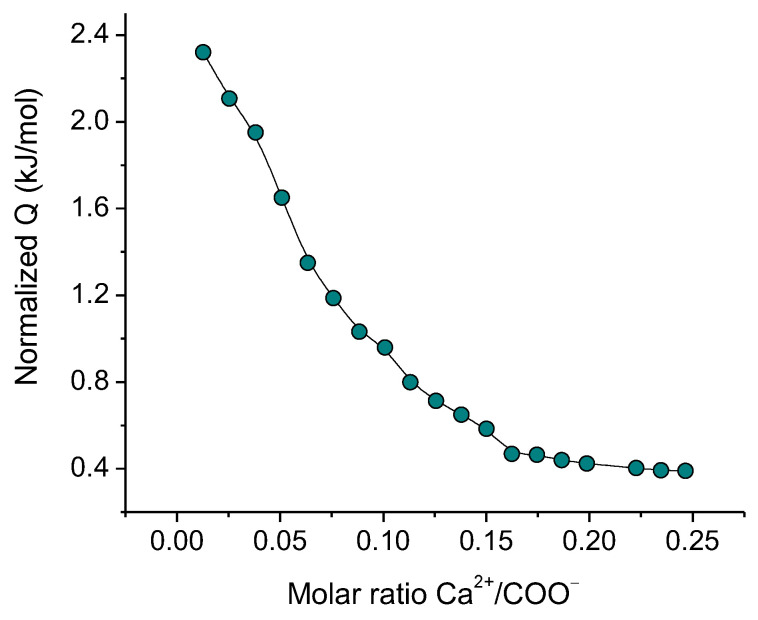
ITC titration curve of 3 wt% CNC dispersion with CaCl_2_ at pH 6.5.

The thermodynamics and stoichiometry of the interaction of Ca^2+^ cations with oppositely charged carboxylate groups of CNCs were studied by ITC. The titration curve obtained is presented in Figure 7. Its fitting with an independent binding site interaction model gives the enthalpy ∆H = 1.9 kJ/mol, the stoichiometric number n = 0.08, the entropy ∆S = 72 J/mol·K and Gibbs free energy ∆G = −20 kJ/mol. These values are close to thermodynamic parameters obtained by ITC for a similar system [4].

These data show that CNCs and Ca^2+^ interact with each other. Their interaction is endothermic (∆H > 0) and is driven by the increasing of the entropy of the system (∆S > 0), which is most likely due to the release of solvated water molecules surrounding the CNCs nanocrystals and the cation, as a result of the CNC-Ca^2+^ interaction. The stoichiometric number indicates that only one carboxylate group out of every ca. 12 carboxylate groups interacts with calcium ions. This is consistent with the data obtained for carboxylated CNCs [4], where the low stoichiometric number was explained assuming that the negative charge is delocalized over the surface of carboxylated nanocellulose. Figure 7 shows that the system becomes fully saturated by bound Ca^2+^ ions at a Ca^2+^/COO^−^ molar ratio of 0.2.

#### 2.4.2. Fourier Transform Infrared (FTIR) Spectroscopy

**Figure 8:**
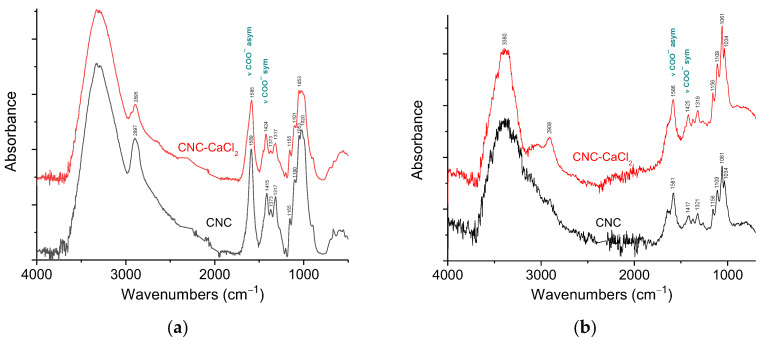
(**a**) ATR-FTIR spectra of CNCs without salt (black) and with 72 mM CaCl_2_ (red) in the dried state. The spectra are offset in the *y*-axis for viewing clarity. (**b**) ATR-FTIR spectra of 6 wt% suspensions of CNCs without salt (black) and with 72 mM CaCl_2_ (red) in water. The spectra are offset in the *y*-axis for viewing clarity.

To further investigate the interaction between CNC carboxylate groups and multivalent cations, we utilized attenuated total reflectance (ATR) FTIR spectroscopy. Figure 8a shows ATR-FTIR spectra of CNCs before and after addition of 0.72 mM CaCl_2_, which corresponds to two calcium ions per one carboxylate group.

The spectrum of CNCs (Figure 8a) contains the main characteristic peaks of cellulose: broad band of stretching vibrations of OH groups (around 3380 cm^−1^), bands of stretching vibrations of C-H bonds (near 2900 cm^−1^), the CH_2_ rocking vibrations (1317 cm^−1^), the C–O–C antisymmetric stretching vibrations at the β(1–4) glycosidic linkage (1155 cm^−1^) and C–O bond stretching vibrations of C2, C3 and C6 carbon atoms linked to OH-groups (1100, 1053 and 1020 cm^−1^, respectively) [28,29,30,31]. Also, the CNC spectrum demonstrates asymmetric and symmetric stretching vibrations of COO^−^ groups at 1589 and 1415 cm^−1^, respectively [32]. No appreciable absorption band of C=O stretching vibration of protonated COOH-groups at ca. 1730 cm^−1^ [30] are detected, confirming that almost all carboxy groups are in the form of carboxylate anions.

From Figure 8a one can see that the most pronounced changes in FTIR spectra of CNCs upon addition of CaCl_2_ concern the bands of stretching vibrations of COO^−^ groups. The band of asymmetric stretching vibrations of carboxylate group is shifted from 1589 to 1585 cm^−1^. Simultaneously, the band of symmetric COO^−^ stretching vibrations demonstrates an even more pronounced shift from 1415 to 1424 cm^−1^. These data suggest that in the interaction with multivalent cations the COO^−^ groups are involved. Another important parameter is the frequency difference Δν between asymmetric and symmetric COO^−^ stretching vibrations, which can be used to identify the bonding mechanism [33]. In the current system, the Δν value is equal to 174 cm^−1^ in pristine CNCs and to 161 cm^−1^ after addition of CaCl_2_; that is, the Δν value decreases by 13 cm^−1^. This is a rather significant change taking into account that according to ITC, the fraction of COO^−^ groups interacting with calcium ions is rather low. Recently, similar shifts were observed in carboxylated cellulose nanofibers/alginate films upon addition of calcium ions [34].

Figure 8b shows ATR-FTIR spectra of CNC before and after addition of 0.72 mM CaCl_2_ in water. In the spectra, H_2_O bending vibrations near 1640 cm^−1^ [35] overlap with the band of asymmetric stretching vibrations of the carboxylate group. But one can observe that in a water-swollen state, the asymmetric COO^−^ stretching vibrations band is shifted from 1581 cm^−1^ to 1586 cm^−1^. At the same time, the band of symmetric COO^−^ stretching vibrations undergoes a significant shift from 1417 to 1425 cm^−1^, similar to the corresponding band in water-free sample (Figure 8a). Thus, the FTIR data indicate the binding of calcium ions to COO^−^ groups.

### 2.5. Isotropic-to-Nematic Transition

CNCs in suspensions can undergo liquid crystalline ordering [36]. They have two characteristic concentrations: φ_1_ and φ_2_. At CNC concentrations below φ_1_, the rods are disordered and the suspension is isotropic. When the CNC concentration falls within the range between φ_1_ and φ_2_, a two-phase system forms, where the isotropic phase is in equilibrium with the chiral nematic domains, in which the rods align in parallel planes that slightly rotate relative to their neighboring planes [37]. At concentrations above φ_2_, the nematic structure extends throughout the entire system, and the suspension becomes completely anisotropic. The critical concentration and the width of the biphasic region depend on the dimensions of and charge on the CNCs [38] and on the ionic strength of the aqueous medium [36].

**Figure 9:**
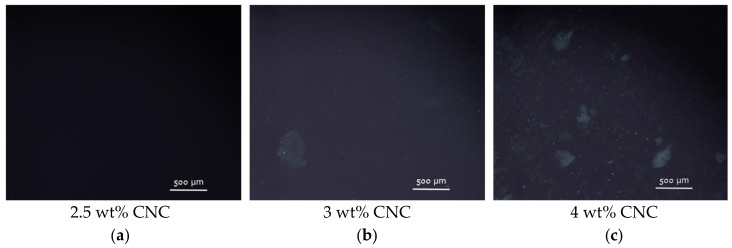

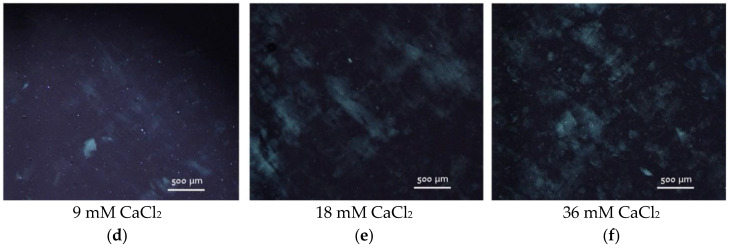
(**a**–**c**) Polarized optical microscopy images of CNC suspensions with different concentrations of nanocrystals: 2.5 wt% (**a**), 3 wt% (**b**) and 4 wt% (**c**); (**d**–**f**) polarized optical microscopy images of 3 wt% aqueous suspensions of CNCs with different concentrations of added CaCl_2_: 9 mM (**d**), 18 mM (**e**) and 36 mM (**f**).

In the present system, polarized optical microscopy (POM) studies reveal the appearance of small chiral nematic domains (tactoids) dispersed in an isotropic continuous phase at a CNC concentration of 3 wt% (2 vol%) (Figure 9b), which suggests the limit of isotropic phase stability φ_1_ = 3 wt%. The same φ_1_ concentration was observed by Shafiei-Sabet et al. for sulfonated CNCs with similar dimensions (100 nm in length L and 7 nm in width D) [13]. According to the Onsager theory [39], the isotropic-to-nematic transitions in the suspension of uncharged rigid rods is expected at volume fraction of rods φ_1_ ≈ 3.34D/L = 0.17, which corresponds to the *w*/*v* fraction 0.25. In charged rods, the transition is shifted to lower volume fraction because of the electrostatic repulsion between them. Due to the charge-induced electric double layer, the actual excluded volume for charged rods is higher than the rod volume. To describe the case of charged rods, Onsager proposed to use an effective diameter D_eff_ > D that mimics the hard core plus soft repulsion between the rods [39]. To estimate D_eff_, the Stroobants−Lekkerkerker−Odijk approach [40] is widely used [41]. For instance, it was shown [42] that D_eff_ becomes 60 nm for 7 nm diameter rods in the solution with ionic strength of 1 mM, which results in the onset of liquid crystal concentration of 2.24 vol%, which is consistent with the results obtained in this paper as well as with some previous experimental data [11,13,36]. At the same time, in contrast to the above-mentioned data [11,13,36], we do not observe any macrophase separation at the isotropic-to-nematic transition. This can be explained by the high charge density of CNCs under study. Indeed, recently it was shown [43] that with increasing surface charge density of CNCs from 0.116 to 0.337 e/nm^2^ the onset of phase separation shifts from 0.5 to 5.1 wt%, whereas in the present system the surface charge density is as high as 1.3 e/nm^2^.

Thus, the initial system used for the gel formation (3 wt% CNCs without salt) possessed only the first signs of nematic ordering (few chiral nematic domains in an isotropic continuous phase) (Figure 9b). When more than 9 mM CaCl_2_ was added, a marble-like texture is observed by POM (Figure 9d–f) in the whole sample volume, similar to that described for sulfonated CNCs (4 wt%) upon addition of 50 mM of different salts (NaCl, CaCl_2_, MgCl_2_, SrCl_2_, AlCl_3_) [14]. Thus, the addition of salt favored the nematic ordering in CNC suspensions, which is in accordance with previously described data [14,15]. This leads to the formation of birefringent gels.

## 3. Conclusions

In the present paper, by several complementary techniques we demonstrate that CNC gel formation upon addition of divalent calcium ions is due not only to the screening of electrostatic repulsion by salt favoring CNC self-association into a network structure but also to crosslinking of nanocrystals as a result of electrostatic binding of divalent cations simultaneously to different CNCs. FTIR data show that calcium ions induce a shift of the bands of asymmetric and symmetric stretching vibrations of CNC carboxylate groups, indicating that these groups are involved in the interaction with calcium ions. The ITC data demonstrate that the CNC-Ca^2+^ interaction is endothermic, being driven by the increase in entropy, which is most likely due to the release of water molecules surrounding the interacting groups: COO^−^ and Ca^2+^. Cryo-ET was first applied to reveal the structure of calcium-crosslinked CNC hydrogel. Cryo-ET together with cryo-EM showed that inside the hydrogels, some of CNCs are aggregated either in short and thin bundles or in long and thick fibrillar-like aggregates. The length and the thickness of the aggregates increase upon addition of CaCl_2_, which can stiffen the CNC network. According to POM data, the gels thus obtained are birefringent.

We performed the cyclic tests to demonstrate the reversibility of CNC gelation and to estimate the recovery time and percent recovery as a function of calcium ion concentration. The observation that the CNC network can be easily destroyed by shear and then quickly reformed if the concentration of calcium ions is high enough opens up the perspectives for the application of such green, renewable and sustainable hydrogels with low environmental impact in many areas, including 3D extrusion printing and the preparation of injectable hydrogels, among others.

## 4. Materials and Methods

### 4.1. Materials

Carboxymethylated CNCs from Cellulose Lab (Fredericton, NB, Canada), and calcium chloride (purity > 97%) from Sigma-Aldrich (St. Louis, MO, USA) were used as received. According to the information provided by the supplier, the CNCs have density ρ of 1.5 g/cm^3^, degree of crystallinity of 86–90%, zeta potential of −40 mV and content of charged COO^−^ groups of 1.2 mmol per gram of CNCs. The average size of a single CNC is 90 nm in length L and 5 nm in width D as determined by cryo-EM (Figure 6). The surface charge density of the CNCs ψ was estimated to be approximately 1.3 e/nm^2^ by dividing the number of charged groups on one nanocrystal by the surface area of that nanocrystal:

$$\psi =\frac{qN_{A}\rho D^{2}L}{2\left(D^{2}+2DL\right)}$$

The average surface area of a nanocrystal was calculated as 2 (D^2^ + 2 DL), assuming that a CNC has a rectangular parallelepiped shape with a height of L = 90 nm (the length of CNC) and a width of D = 5 nm (the width of CNC). The number of charged groups on the surface of one nanocrystal was estimated by multiplying the number of moles of charged COO^−^ groups per gram of CNC q by the Avogadro number N_A_ and the mass of one CNC, ρV, where ρ is the density of the CNC and V is its volume (V = D^2^L). Our elemental analysis data demonstrate that the CNCs do not contain sulfur.

All solutions were prepared using distilled, deionized water that had been purified by the Millipore Milli-Q system.

### 4.2. Preparation of CNC Suspensions and Hydrogels

First, 3 wt% CNC suspension was prepared by dispersing CNC powder in water by ultrasonic treatment (5 min, 100 W) with ultrasonic homogenizer Sonics VCX-500 Vibra-Cell (Cole Palmer, Vernon Hills, IL, USA). Then a CaCl_2_ salt solution was added and mixed with an Ultra-Turrax homogenizer IKA (Staufen, Germany) for 15 min at 7000 rpm and then with a magnetic stirrer for 1 h at 250 rpm. The prepared samples were left for 1 day for equilibration.

### 4.3. Rheometry

The rheological studies were performed on a Physica MCR 301 rheometer (Anton Paar, Graz, Austria) using two different measurement cells: a plate–plate cell with a diameter of 25 mm and a gap of 1000 μm, and a cone–plate cell with a diameter of 40 mm and cone angle 2°. Prior to each experiment, the samples were allowed to reach equilibrium state after loading in the cell for 10 min. The temperature was kept at 25 °C with a Peltier element. In oscillatory shear tests, the frequency dependencies of the storage G′ and loss G″ moduli were measured in the frequency range from 0.04 to 30 rad/s at a controlled deformation of 1% (within the linear viscoelastic regime as determined by performing amplitude sweep experiments at a frequency of 1 rad/s) as reported previously [44].

### 4.4. Cryogenic Electron Microscopy and Cryogenic Electron Tomography

The measurements were carried out on a Titan Krios 60-300 TEM/STEM (Thermo Fisher Scientific, Waltham, MA, USA) CryoEM, equipped with a K3 direct electron detector (Gatan, Pleasanton, CA, USA), a BioContinuum imaging filter (Gatan, Pleasanton, CA, USA) and a Cs corrector (CEOS, Heidelberg, Germany), at an accelerating voltage of 300 kV.

Lacey EM grids (Ted Pella, Northport, NY, USA) were glow-discharged for 30 s at 0.26 mbar using a current of 20 mA with a PELCO easiGLOW (Ted Pella, Northport, NY, USA). In the vitrification procedure, 0.5 µL of the sample solution were applied to Lacey EM grids. The grids were then blotted for 2.5 s and allowed to relax for 5 s using a Vitrobot Mark IV (Thermo Fisher Scientific, Waltham, MA, USA) at 20 °C and 100% humidity. Just after the relaxation step, the grids were quickly plunged into liquid ethane. Details of the experiments and data analysis are reported elsewhere [45].

Tilt series with a dose-symmetric tilt scheme were obtained using Serial EM 4.0.28 software [46]. The tilt range was from −60° to +60° with 3° increments, defocus ranging from −8 to −6 µm. Five frames were collected for each image, resulting in a total dose of 60 e-/Å^2^ per tilt series. The tilt series were acquired at a nominal magnification of 26,000 times, resulting in a pixel size of 2.68 Å, with the post-column energy filter set to a slit width of 20 eV. Frames were aligned using Warp 1.0.9 software [47]. Tilt series were aligned using a fiducial-less patch tracking method, and tomograms were reconstructed by using back projection with a SIRT-like filter in IMOD 4.11 [48]. The tomograms were binned by four, resulting in a pixel size of 10.72 Å. The isotropic resolution of the tomograms was improved using the IsoNet 0.2 software [49]. The final visualization was conducted in Avizo (Thermo Fisher Scientific, Waltham, MA, USA).

### 4.5. Isothermal Titration Calorimetry

Calorimetric experiments were performed using a Nano ITC isothermal calorimeter (TA Instruments, New Castle, DE, USA). A 3 wt% CNC suspension was prepared and sonicated for 5 min at 100 W and then placed in the calorimetric cell. A 100 mM CaCl_2_ solution was loaded into a syringe, and 20 injections of 2.5 μL each were added to the ITC sample cell containing 0.65 mL of CNC suspension, with a 20 min time interval between injections. A blank experiment to estimate the heat of dilution was conducted by injecting CaCl_2_ into 0.65 mL distilled water, and the data were used to correct for the heat of dilution by subtracting the blank from the CNC experiment data. A thermodynamic profile of binding interaction was determined by fitting the data to an independent binding site interaction model.

### 4.6. Fourier Transform Infrared Spectroscopy

FTIR spectra were measured with Bruker Vertex 70v FTIR spectrometer (Ettlingen, Germany) in 4000–400 cm^−1^ range with a resolution of 4 cm^−1^ in ATR mode using a PIKE GladyATR device (Pike Technologies, Madison, WI, USA) with diamond ATR unit. Spectra for aqueous suspensions were background corrected using water spectrum [50].

### 4.7. Polarized Optical Microscopy

POM measurements were carried out using a polarized light microscope Nikon Eclipse LV100 POL (Tokyo, Japan) with a CFI TU Plan Fluor Epi objective lens (p 5×), at room temperature, for suspensions or gels placed in the space (0.3 mm) between two glass plates.

## Figures and Tables

**Table 1:** Recovery time and percent recovery at periodic variation of shear rate for aqueous suspensions containing 3 wt% CNCs and different concentrations of CaCl_2_.

Concentration of CaCl_2_, mM	Recovery Time, s		Percent Recovery	
	Second Cycle	Third Cycle	Second Cycle	Third Cycle
18	59.4	162.3	41	30
36	9.7	10.6	49	44
72	15.7	15.8	91	87

## Data Availability

The original contributions presented in this study are included in the article. Further inquiries can be directed to the corresponding author.
